# Defective States in Micro-Crystalline CsPbBr_3_ and Their Role on Photoconductivity

**DOI:** 10.3390/nano9020177

**Published:** 2019-02-01

**Authors:** Mara Bruzzi, Fabio Gabelloni, Nicola Calisi, Stefano Caporali, Anna Vinattieri

**Affiliations:** 1 Dipartimento di Fisica e Astronomia, Università degli Studi di Firenze, Via G. Sansone 1, Sesto Fiorentino, 50019 Firenze, Italy; fabio.gabelloni@unifi.it (F.G.); anna.vinattieri@unifi.it (A.V.); 2 Istituto Nazionale di Fisica Nucleare, Sezione di Firenze, Via G. Sansone 1, Sesto Fiorentino, 50019 Firenze, Italy; 3 Dipartimento di Ingegneria Industriale, Università degli Studi di Firenze, Via S. Marta 3, 50139 Firenze, Italy; nicola.calisi@unifi.it (N.C.); stefano.caporali@unifi.it (S.C.)

**Keywords:** CsPbBr_3_, microcrystals, defects, thermally stimulated current, charge transfer

## Abstract

Intrinsic defects in CsPbBr_3_ microcrystalline films have been studied using thermally stimulated current (TSC) technique in a wide temperature range (100–400 K). Below room temperature, TSC emission is composed by a set of several energy levels, in the range 0.11–0.27 eV, suggesting a quasi-continuum distribution of states with almost constant density. Above room temperature, up to 400 K, the temperature range of interest for solar cells, both dark current and photocurrent, are mainly dominated by energy levels in the range 0.40–0.45 eV. Even if measured trap densities are high, in the range 10^13^–10^16^ cm^−3^, the very small capture cross-sections, about 10^−26^ m^2^, agree with the high defect tolerance characterizing this material.

## 1. Introduction

The increasing interest of the scientific community on perovskite materials for a wide range of applications as, e.g., solar cells, light-emitting diodes, lasers, photodetectors, is motivated by their favorable properties, such as tunable band gap, strong optical absorption, ambipolar charge transport, and long electron-hole diffusion lengths [1,2,3]. All-inorganic perovskites CsPbX_3_ (X = Cl, Br, and I) have been recently proposed due to their high chemical stability [4], in the form of single crystals as well as nanocrystals [5,6] and microcrystalline thin films [7,8]. Early electrical conductivity studies showed that the main electrical parameters of the investigated samples suffered from the presence of defects. Indeed, it is in general well known that defects, even in small concentrations, can significantly deteriorate the transport properties of a semiconductor material and the related device parameters [9]. Therefore, defects should be characterized, in detail, to get a thorough knowledge of the whole device performance. Up to now, the study of defects in inorganic perovskites is still limited mainly to theoretical evaluations [10]. Experimentally, the presence of defects in perovskites has been often inferred indirectly by simulating the current–voltage characteristics of perovskite solar cells [11,12,13,14,15,16,17]. Nonetheless, a direct investigation of electrically active defects in semiconductor material should be performed on the bare semiconductor layer, excluding the other components of the cell. This is generally carried out by means of thermal spectroscopy techniques such as deep level transient spectroscopy (DLTS), photo-induced current transient spectroscopy (PICTS), and thermally stimulated current (TSC); being much more sensitive than opto-physical techniques, they are able to detect even very low concentrations of defects [18]. DLTS and PICTS monitor rapid changes of the capacitance/photoconductivity after repeated electrical/optical pulses carried out during the thermal scan. In a TSC, the priming process is performed only once, at low temperature, either applying an electric pulse or illuminating the sample with an above-bandgap radiation. Then, after filling, the temperature is increased with a constant heating rate and the current peak due to electron/hole emission from traps is measured. Peak parameters such as maximum temperature, T_max_, peak intensity, I_max_, and peak FWHM, can be used to estimate the energy level position in the forbidden gap, E_t_, the trap capture cross-section, σ, and the concentration of the trap, N_t_. TSC is quite useful as it can be applied to any kind of electric field profile settled within the sample, including the ohmic configuration. On the contrary, DLTS, monitoring changes in the capacitance, can be used when either a Schottky barrier or a p–n junction is present. Moreover, DLTS is based on the assumption that shallow donor/acceptors have a much higher concentration than deep traps, a thing, in general, not occurring in wide bandgap semiconductors, often carrying high concentrations of deep energy levels from native, intrinsic defects. Moreover, fast pulses used in a DLTS/PICTS analysis are, in general, not able to saturate the trap occupation, so they can be used only to evaluate the energy level and the capture cross-section of the trap, not its concentration. A few works using thermally stimulated current on perovskites have been published recently, focusing, in particular, low temperature ranges, below 300 K [19,20]. Nonetheless, due to their potential use in photovoltaic devices, the temperature range of interest for perovskite-based solar cells could easily reach 50–60 °C [21], a temperature range not yet investigated. In [20], TSC spectra were analyzed using SIMultaneous-Peak Analysis (SIMPA) in the assumption that any measured spectral emission could be given by an arbitrary sum of components from a multitude of energy levels acting independently of each other. As a result, single isolated TSC emissions have also been often interpreted as the result of the sum of several, not-resolved TSC peaks. Nonetheless, an accurate TSC analysis should spend efforts in separating possible overlapping components and this is, in general, achieved by cross-correlating results obtained by varying parameters such as heating rate, filling time, filling temperature, e.g., using heating-rate (β-)variation and delayed-heating methods [18].

In this work, photoconductivity and TSC have been carried out in the temperature range 100–400 K, to demonstrate the presence of native defects and their influence on the electrical photoresponse of CsPbBr_3_. Heating-rate variation and delayed-heating methods have been applied to investigate possible overlapping components occurring in TSC emissions.

## 2. Materials and Methods

**Figure 1:**
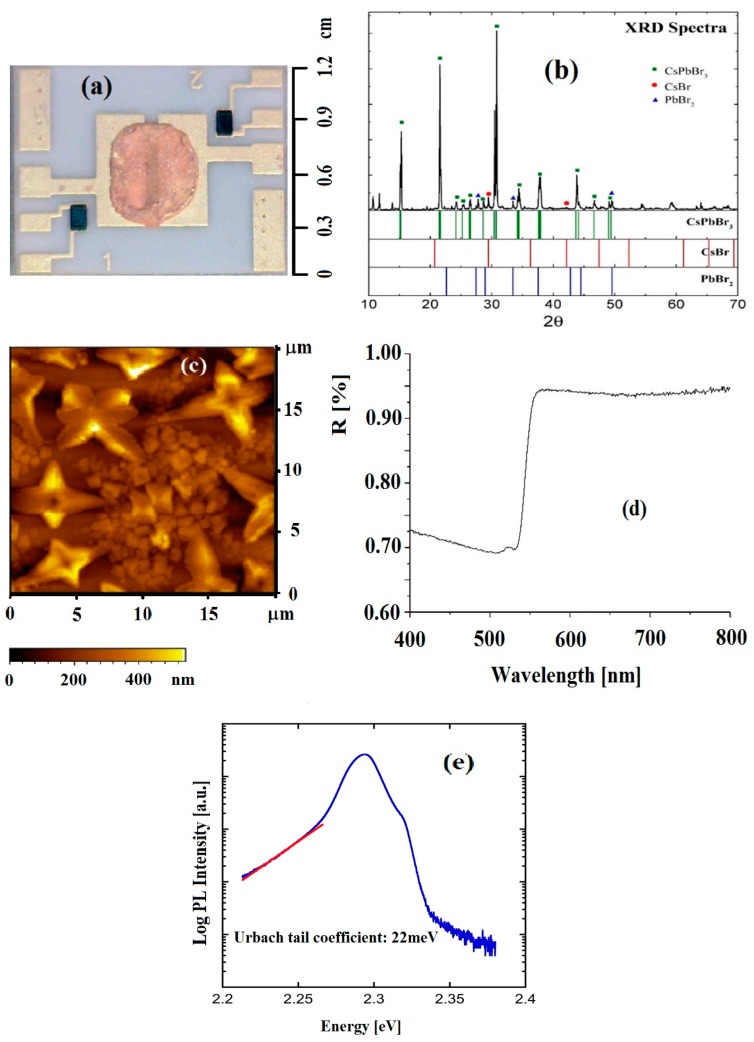
(**a**) Perovskite film, deposited on an alumina substrate having two parallel gold contacts, an integrated heater on the back side, and two symmetrical Pt sensors on lateral sides. (**b**) XRD spectra of the deposited material. (**c**) AFM image of the perovskite film showing its microcrystalline nature; (**d**) reflectance spectrum of the same film; (**e**) PL spectrum of a similar drop-casted sample deposited on a glass substrate, with the exponential fit showing a 22meV Urbach tail coefficient.

CsPbBr_3_ films were deposited by drop-casting directly on an alumina printed circuit board (PCB) especially designed for electrical tests of thin semiconductor films at a wide temperature range [22]. The PCBs have two/four parallel gold contacts, 7 mm long, spaced at 0.8 mm, as well as integrated Pt temperature sensors and a heater on the back side. A picture of a perovskite film deposited on a two-contact PCB is shown in Figure 1a, where the gold contacts have a thickness of about 20 μm. Planar geometry with electrodes placed under the perovskite film, which is rather thick, dense, and covers the overall pin-contacts area, prevents instability of the electrical conductivity due to surface oxidization, resulting in stable and reproducible electrical conductivity measurements, also avoiding surface damage due to, e.g., scratches made by spring probes on top of the soft structure of the perovskite. The film has been obtained starting from CsBr and PbBr_2_, mole ratio 1:1, in a saturated dimethyl sulfoxide (DMSO) solution. Chemicals were purchased from Sigma-Aldrich and used without further purification. Cesium bromide was synthetized adding bromidic acid to cesium carbonate until cessation of bubbling. The obtained solution was dried and the solid was washed several times with acetone. A white crystalline powder was obtained and characterized with XPS and XRD to confirm the formation of cesium bromide. After thermal annealing at 150 °C, a dense and continuum layer of interconnected CsPbBr_3_ microcrystals of the order of a few μms was manufactured. The film has been then covered with polymethylmethacrylate (PMMA) to prevent deterioration due to air and water vapor [23]. XRD (Figure 1b) and XPS analyses demonstrated the good quality of the film with no residual solvent and contaminants. The AFM micrograph (Figure 1c) shows microcrystals agglomerating with a typical size up to 5–10 μm. A reflectance spectrum obtained for this film is shown in Figure 1d. A drop-casted film prepared with the same procedure was tested by photoluminescence spectroscopy (PL) to study its spectral characteristics, and the plot of Figure 1e shows an high quality of the emission, similar to literature data [24].

The PL emission is dominated by bound exciton recombination at 2.3 eV and the free exciton contribution at 2.32 eV appears like a shoulder in the high energy side. The exponential tail in the low energy side comes from strongly localized emission. The red line is an exponential fit providing an Urbach tail coefficient of ≈ 22 meV, similar to values reported in literature for CsPbBr_3_ (see, for instance, [25]). The Urbach tail shows the presence of high energy trap states which, nevertheless, do not play a major role at room temperature given the efficient thermal activation.

Current–voltage (I–V) characteristics have been measured in a planar two-point probe configuration by means of a Keithley 6517 high resistance source/meter at room temperature. In the dark, the I–V behavior is linear in the range 0–10V, evidencing an ohmic regime, symmetric when reversing the voltage, and with negligible barriers at contacts. A resistivity of about 1 GΩcm was measured in dark at room temperature, in good agreement with the literature [26]. Taking into account the forbidden gap (Eg, ~2.3 eV) and of the effective masses reported in literature for this material [27], the intrinsic resistivity should be several orders of magnitude higher than the measured value. As a consequence, unintentional doping occurs in the material, due to native defects in the bulk and/or at grain boundaries. To assess the type of this unintentional doping, Hall effect measurements were performed at room temperature on films deposited on four squared contact PCBs. We used a Keithley 6430 as the current source, a Keithley 2182 nanovoltmeter for voltage readout, and a B = 0.55 T magnet (from ECOPIA, Korea). A positive value of the Hall coefficient R_H_ ~ 10^10^ cm^3^/C has been measured in the dark, indicating p-type conductivity with a Hall mobility $\mu_{H}=\frac{R_{H}}{r_{H}\rho }$ ~ 10 cm^2^/(Vs), in agreement with the recent literature [26].

Photoconductivity (PC) and TSC measurements have been carried out at constant voltage, at several temperatures in the range 150–400 K. Measurements below room temperature were carried out placing the sample-holder in a dewar partially filled with liquid nitrogen. The sample heater was biased by a TTi QL564P power supply, and temperature was read out by either a Keithley 2001 electrometer or DRC-91C Lakeshore temperature controller. The overall system was controlled via MATLAB Toolbox software. Priming was performed using a white LED and 400 nm LED/laser sources with power up to 0.8 mW. During the filling process at fixed temperature, the sample was illuminated up to several minutes while monitoring the current. Thermally stimulated current (TSC) measurements have been performed after each PC measurement in the temperature range 150–400 K. After the filling process, temperature was increased slowly, from the initial value T_in_ to the final temperature T_fin_, using a fixed and constant heating/cooling rate, β, chosen in the range 0.05–0.25K/s. The current has been monitored during the entire cycle of the heating stage up to T_fin_ and, afterwards, (cooling stage) back to T_in_.

To isolate the component due to charged carriers emitted by traps towards the conduction/valence bands during the heating scan, a TSC curve is calculated as the difference of the current measured, at same temperature, during the heating and the cooling stages [18]. Estimate of the resistivity of the sample in dark, as a function of the temperature, has been performed from the current measured in the cooling stage.

## 3. Numerical Analysis

### 3.1. Photoconductivity

Current flowing in an n-type semiconductor equipped with two ohmic contacts is given by

(1) $$I={\mathrm{qnv}}_{d}A$$

with q is electron charge absolute value, A surface normal to the electric field F, v_d_ = μ_n_ F, is the drift velocity (F = V_b_/d, with V_b_ potential drop at electrodes, d is distance between electrodes), μ_n_ electron mobility. During an illumination pulse, the electron concentration n changes following the rate equation given below:

(2) $$\frac{\mathrm{dn}}{\mathrm{dt}}=G-\frac{n}{\tau }-\frac{{\mathrm{dn}}_{t}}{\mathrm{dt}},$$

where G is the generation rate, τ the recombination lifetime at recombination centers, dn_t_/dt is the rate of change of the occupied traps concentration, with N_t_ being their total concentration. The trap occupation rate can be written as [18]

(3) $$\frac{{\mathrm{dn}}_{t}}{\mathrm{dt}}=c_{n}\left(N_{t}-n_{t}\right)-e_{n}n_{t},$$

with c_n_ = σv_th_n capture coefficient, and $e_{n}={\sigma v}_{\mathrm{th}}N_{c}e^{\frac{E_{t}-E_{c}}{\mathrm{KT}}}$ emission coefficient. Here, σ is the capture cross-section of the defect, v_th_ is the thermal velocity, $v_{\mathrm{th}}=\sqrt{\frac{3K_{B}T}{m^{*}}}$, and m* effective mass. The concentration of free carriers, n, depends on the effective concentration at minimum of conduction band N_c_, and on the quasi-Fermi level position $E_{\mathrm{Fn}},$ with respect to the minimum of the conduction band, E_c_, through the relationship $n=N_{c}e^{\frac{E_{\mathrm{Fn}}-E_{c}}{\mathrm{KT}}}$.

Our numerical approach for the analysis of the photoconductivity is the following. We assume the presence of a set of defects, each characterized by energy level E_t_, a capture cross-section σ, and a concentration N_t_, plus a recombination center with recombination lifetime τ. Each emission coefficient, e_n_, is then determined by the respective values of E_t_ and σ: they are constant parameters as the temperature is fixed during the photoconductivity measurement. Conversely, each capture coefficient, c_n_, is changing during the illumination pulse; it can be determined directly from the measured current, I: $c_{n}=\frac{{\sigma v}_{\mathrm{th}}I}{{\mathrm{qv}}_{d}A}$. Knowing them, first, we evaluate the trap occupancy before the pulse, when G = 0, $\frac{{\mathrm{dn}}_{t}}{\mathrm{dt}}=0,\;\frac{\mathrm{dn}}{\mathrm{dt}}=0$. In this way, from Equation (3), we get the trap occupancy of each trap: $\frac{n_{t}}{N_{t}}=\frac{c_{n}}{c_{n}+e_{n}},$ constant before the pulse. When the illumination starts, with known G ≠ 0, constant during the pulse, the trap occupancy changes. We can determine its value by iteration, starting from the initial trap occupancy before the pulse. Iteration can be continued after the pulse when, again, G is null. Knowing the change of n_t_ with time it is possible to infer the change of n with time from Equation (2) and so we estimate the current, I_fit_, to be compared with that experimentally measured, I. Opportune changes of the set of trap parameters are then chosen to finally best-fit (χ-square method) the measured current, I, to the one determined numerically, I_fit_.

### 3.2. Thermally Stimulated Current

When the light is switched off after the illumination pulse (G = 0), the main operative process is thermal emission, as c_n_ << e_n_ (assumption of negligible retrapping). Note that during the thermally stimulated current measurement, carried out when the sample is heated with a constant heating rate after switching off the light pulse, the emission coefficient e_n_ is no longer a constant. Equation (2) reduces to $\frac{{\mathrm{dn}}_{t}}{\mathrm{dt}}=-e_{n}n_{t}$. The trap occupation can be written

(4) $$n_{t}=n_{t0}{\;e}^{-\int_{t_{i}}^{t}e_{n}\left(t\right)\mathrm{dt}}=n_{t0}e^{-\frac{1}{\beta }\int_{T_{i}}^{T}e_{n}\left(T\right)\mathrm{dT}},$$

having assumed a constant heating rate β = dT/dt. The electrons released from traps are collected at electrodes within an effective lifetime τ_eff_, so that [9]

(5) $$I\left(t\right)={\;q\;A\;F\;\mu }_{n}\tau_{\mathrm{eff}}{\;e}_{n}{\;n}_{t},$$

and the thermally emitted current is

(6) $$I_{\mathrm{TSC}}\left(T\right)={e\;\mu }_{n}{\;F\;S\;\tau }_{\mathrm{eff}}{\;n}_{t0\;}e_{n}\left(T\right){\;e}^{-\frac{1}{\beta }\int_{T_{i}}^{T}e_{n}\left(T\right)\mathrm{dT}}.$$

Using this expression (first order kinetics) Chen [28] showed that one can obtain an estimation of the energy level associated to the peak, E_t_, considered as a single, component, from the expression

(7) $$E_{t}=c\frac{K_{B}T_{\max}^{2}}{\delta }-b\left(2K_{B}T_{\max}\right),$$

where c = 1.51, b = 1.58, δ = T_max_ − T_1_, T_max_ peak temperature, and T_1_ is the lowest temperature of the peak FWHM. To get an estimation of the energy level and of the capture cross-section of the trap, the β-variation method can also be applied. Here, a set of TSC measurements with different constant heating rates β and same filling procedure is carried out. The temperature where the TSC peak occurs, T_max_, is recorded at each β. For the same energy level and cross-section pair, by increasing the heating rate, the peak temperature will increase, following the relationship [18]:

(8) $$\ln\left(\frac{T_{\max}^{4}}{\beta }\right)=\frac{E_{t}}{K_{B}T_{\max}}+\ln\left(\frac{E_{t}}{{\sigma K}_{B}\gamma }\right).$$

It is then possible to determine the energy level related to the defect responsible for the TSC peak from the slope of the linear trend and the capture cross-section of the trap from the intercept of Equation (8). Finally, the analysis of the thermally stimulated current can be performed, also numerically, best-fitting data using Equation (6) with proper values of $\tau_{\mathrm{eff}},{\;n}_{t0},$ E_t_, and σ parameters.

## 4. Results

### 4.1. Thermally Stimulated Current

**Figure 2:**
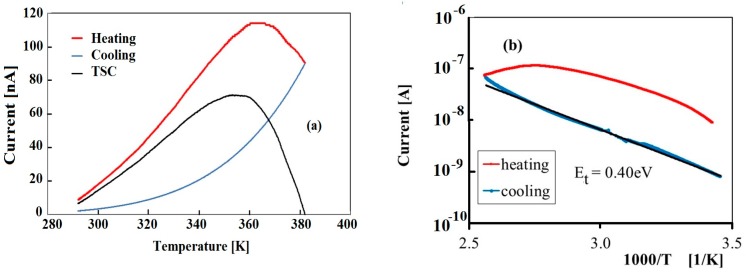
(**a**) Thermally stimulated current measured in the range 300–400 K after priming at T = 291.6 K, V_b_ = 5 V, with a 400 nm LED source. (**b**) Same curve vs. 1000/T, with best fit of the cooling stage evidencing a decay of the current characterized by an activation energy E_t_ = 0.40 eV.

Figure 2a shows the thermally stimulated current measured in the temperature range 300–400 K (heating rate 0.08 K/s) after priming at T = 291.6 K with a white LED source (V_b_ = 5 V). A large peak at about 360 K is observed, with an FWHM of about 55 K. Figure 2b shows the same curves as a function of 1000/T, showing the exponential decay of the current in the cooling stage, characterized by an activation energy E_t_ = 0.40 ± 0.01 eV, as from the fitted tail.

**Figure 3:**
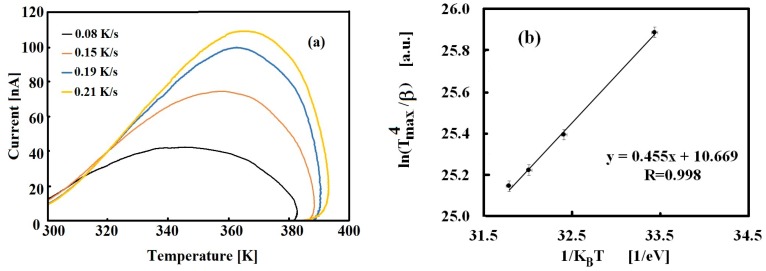
(**a**) Thermally stimulated current (TSC) measured in the 300–400 K range measured with four different heating rates, V_b_ = 5 V, after priming at T = 295 K with a white LED source (same priming conditions). Data are obtained by subtracting the background current measured during cooling from the current measured during the heating stage. (**b**) Linear plot and best-fit used to determine trap energy level and capture cross-section.

Using the Chen expression, Equation (7), we get a first estimation of the energy level associated to the peak, considered as a single component: E_t_ = 0.42 eV. To confirm this estimation, the β-variation method has been also applied. Figure 3a shows the TSC peaks measured with the same filling procedure at room temperature and four different heating rates (cooling current already subtracted from heating current). In the plot, the increase of the peak comes with a shift toward higher temperatures when faster heating rates are used. The plot of $\ln\left(\frac{T_{\max}^{2}}{\beta }\right)\mathrm{vs}\;\frac{1}{K_{B}T},$ shown in Figure 3b, is best-fitted, getting E_t_ = 0.45 eV and σ = 3 × 10^−26^ m^2^.

**Figure 4:**
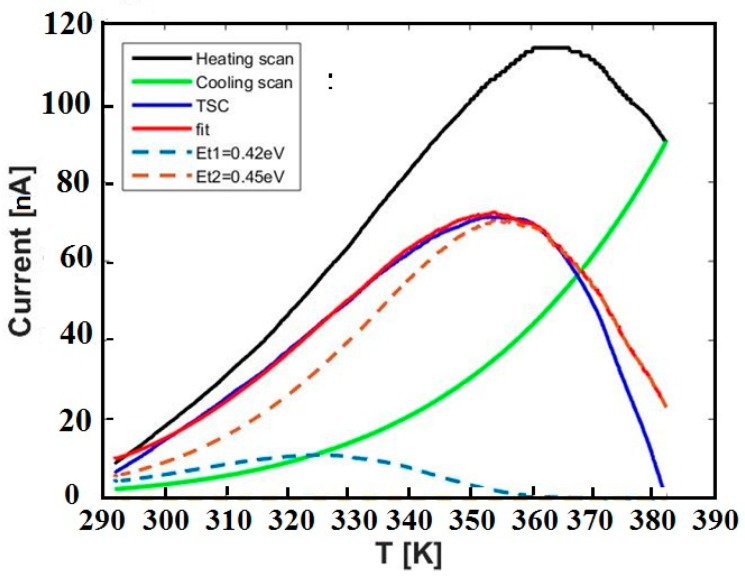
TSC data shown in Figure 2 are, here, compared with the best-fit obtained using standard TSC expression for two discrete levels dominating the stimulated current, with energy as given in the legend.

Analysis of the thermally stimulated current has also been carried out numerically by fitting TSC data to Equation (8). The TSC peak shown in Figure 2a can be nicely fitted considering a dominant component with energy Et = 0.45 eV and capture cross-section σ = 1.5 × 10^−26^ m^2^ and a minor component with E_t_ = 0.42 eV as a shoulder in the lower-T range. Data and best-fit using these parameters are shown in Figure 4.

We conclude that, in the range 300–400 K, TSC is dominated by a very broad emission, due to energy levels at 0.42–0.45eV characterized by very low capture cross-sections, about 1–3 × 10^−26^ m^2^.

**Figure 5:**
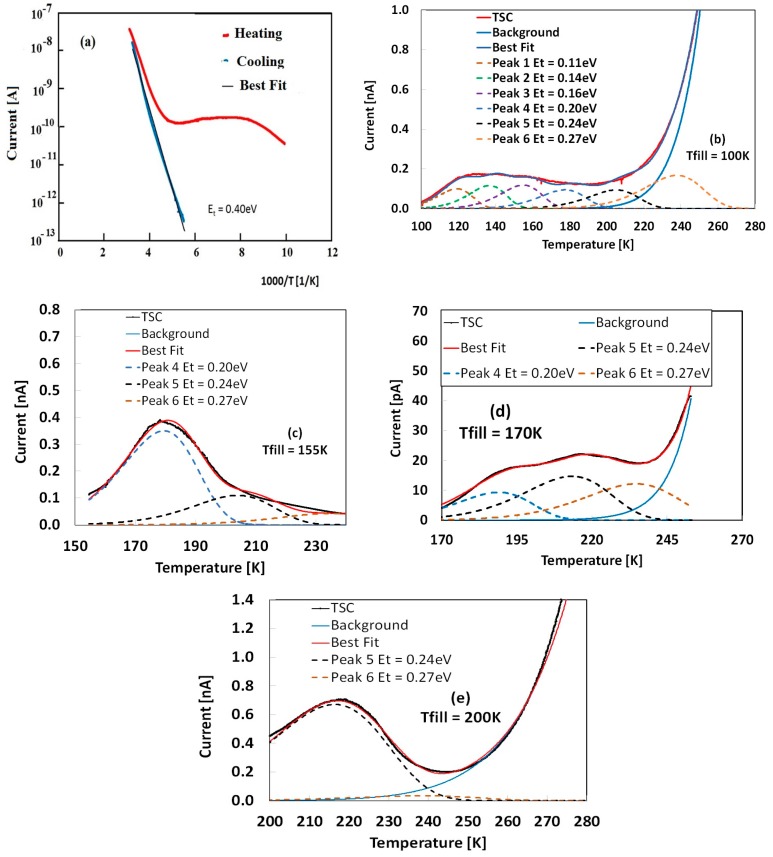
TSC after priming with a 400 nm LED source, V_b_ = 5 V, β = 0.08 K/s. (**a**) T_fill_ = 100 K, cooling scan thermally activated with E_t_ = 0.40 eV. (**b**–**e**) Thermally stimulated current (TSC) obtained with filling at different temperatures T_fill_: (**b**) 100 K; (**c**) 155 K; (**d**) 170 K; (**e**) 200 K. Best-fit of curves shown in (**b**–**e**) made considering background signal (cooling) and same set of defects, as given in legend.

The low temperature range has been investigated, too. TSC measurements have been carried out in the range 100–300 K. Figure 5a shows TSC measurement after priming with an LED source at 400 nm, V_b_ = 5 V, heating rate 0.08 K/s. As with the high temperature range, background current measured during cooling appears thermally activated with energy E_t_ = 0.40 ± 0.01 eV. In the heating stage, we observe a very large TSC emission.

Such a broad TSC signal reveals, once more, the presence of defects with very low capture cross-sections, like those measured in the high temperature range. The TSC emission measured in the range 100–200 K is shown in Figure 5b. Best-fit returns a set of 6 peaks, characterized by energy levels E_t_ = 0.11, 0.14, 0.16, 0.19, 0.24, and 0.27 eV, and capture cross-sections σ ~ 10^−26^ m^2^. Given such a complex structure of defects, uncertainty on the determination of trap parameters can be very high, so we carried out a further inspection, using the delayed-heating method. A set of TSC measurements has been performed with same priming procedure but increasing filling temperature, to progressively decrease emissions from shallower components and focus on ones coming from deepest energy levels. TSC measurements with T_fill_ = 155, 170, and 200 K, are reported in Figure 5c–e.

Results show that the three energy levels with E_t_ = 0.11, 0.14, and 0.16 eV are active only in the range 100–150 K, while energy levels with E_t_ = 0.20 and 0.24 eV, and 0.27 eV, are dominating, respectively, in the ranges 150–200 K and 200–250 K. Consistency of our estimations is enforced by the fact that in all measurements taken with four different T_fill_, the energy levels involved in TSC emissions are the same within an uncertainty of about 0.01 eV. As a matter of fact, energy levels found in this temperature range are very close with each other, almost equally spaced, and with rather similar emission intensity: this suggests a quasi-continuous distribution of levels in the range 0.1–0.3 eV characterized by an almost constant density of states. Finally, Equation (6) allows, also, estimation of the concentration of traps, n_t0_, involved in our TSC analyses. We get a reasonable evaluation considering, as the effective time $\tau_{\mathrm{eff}}$, the transit time of the released charges moving across electrodes in the presence of the electric field F: $\tau_{\mathrm{eff}}=\frac{W}{\mu F}$ ~ 120 μs. In this way, we find values of about 10^16^ cm^−3^ for defects related to the energy levels at 0.42−0.45 eV, and 10^13^–10^14^ cm^−3^ for traps with energy levels in the range 0.11–0.27eV.

### 4.2. Photoconductivity

**Figure 6:**
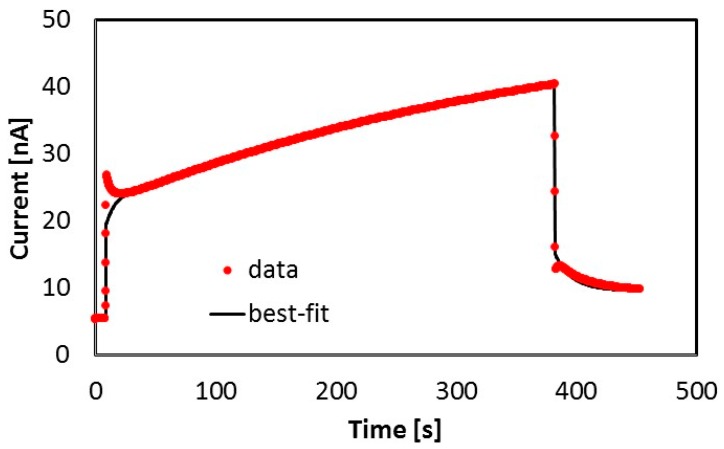
Photoconductivity measured at T = 291.6 K, V_b_ = 5 V with a 400 nm LED source, during the priming used for thermally stimulated current analysis shown in Figure 2.

Figure 6 shows the current measured at room temperature during the filling pulse used for the thermally stimulated current analysis shown in Figure 2.

**Figure 7:**
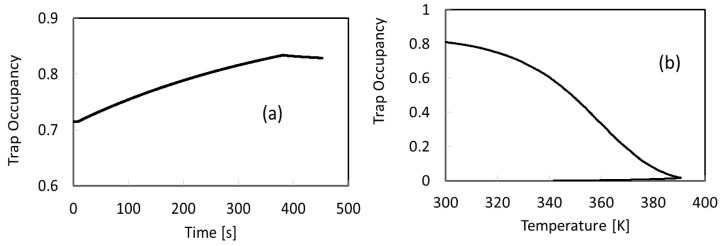
Trap occupancy of the energy level E_t_ = 0.45 eV during (**a**) the photoconductivity measurement shown in Figure 6, and (**b**) the TSC measurement shown in Figure 2 and Figure 4.

The time structure of the current pulse is characterized by slow rise/decay transients during and after illumination, which we assume to be related to defect charging/discharging, superimposed on the primary photocurrent (fast rise/decay components occurring when the pulse is switched on/off), ruled out by the recombination time τ. Best-fitting to the measured photoconductivity has been carried out considering equation 1 and 3, and taking into account results of the thermally stimulated current measurement performed immediately after this filling process. Considering the generation rate during illumination as $G=\frac{\alpha \;I}{h\nu }$, α absorption coefficient, I = light intensity, and ν frequency of the monochromatic incident radiation, we obtain, for a 400 nm wavelength source with 0.3 mW power impinging on a spot with W = 0.8 mm diameter (distance between contacts) a generation rate G ~ 5 × 10^21^ s^−1^cm^−3^. Experimental data are best-fitted considering one dominant trap, with E_t_ = 0.45 eV, σ = 1.7 × 10^−26^ m^2^, and n_t0_ ~ 3 × 10^16^ cm^−3^ capturing and releasing carriers. These results are in very good agreement with those obtained best-fitting the successive TSC measurement, shown in Figure 2 and Figure 4. Hence, both room temperature photoconductivity and TSC at high temperature can be explained considering the 0.45 eV energy level capturing and increasing its trap occupancy during illumination, and then releasing its charge during the TSC process, down to an almost complete discharge. Trap occupancies of this dominant defect during the photoconductivity and TSC measurements are shown, respectively, in Figure 7a,b.

## 5. Discussion

CsPbBr_3_ microcrystalline films deposited by drop-casting have been studied by means of thermally stimulated current (TSC) technique, to detect intrinsic electrically active defects and study their role on dark current and photocurrent in a wide temperature range (100–400 K). Several broad TSC emissions have been observed after priming with 400 nm light pulses. Data analyses have been carried out with several methods to identify energy levels, capture cross-sections, and trap concentrations: a set of energy levels in the range 0.11–0.45 eV have been determined. Even if trap densities are high, up to 10^16^ cm^−3^, with defects probably mainly concentrated at microcrystalline boundaries, the very small capture cross-sections of defects measured by us, about 10^−26^ m^2^, is able to significantly reduce their impact on the optoelectronic performance of lead halide perovskite, a high defect tolerance property already evidenced in [10]. Dark current appears to be thermally activated with an energy around 0.40 eV, in the overall investigated temperature range. Below room temperature, TSC emission is dominated by a quasi-continuum distribution of energy levels in the range 0.11–0.27 eV, with an almost constant density of states while, above room temperature, up to 400 K, detected energy levels are at 0.42–0.45eV. Rise and decay times of the photoconductivity at room temperature can be described as dominated by a trap at 0.45eV, in very good agreement with TSC analysis. A tentative assignment to lattice defects in CsPbBr_3_ can be performed considering first-principle calculations [10]. Here, a group of shallow energy levels, in the range 0.1–0.2 eV from the valence/conduction band, are associated to vacancy and interstitials, V_Cs_, V_Pb_ (acceptors) V_Br_, Csi (donors), as well as to antisite defects such as Cs_Br_, Pb_Cs_, (donors), and Br_Cs_ (acceptor). A second group, with intermediate energy levels in the range 0.35–0.65 eV, are assigned to Pb_i_ and Pb_Br_ (donors). Deepest energy levels, 1.0 and 1.4 eV, relate to two different charged states of the Br_Pb_ antisite defect (acceptors). Unfortunately, TSC analysis is not able to distinguish between electron- and hole-like traps, as released electrons and holes give a contribution of the same sign to the total current. Nonetheless, our measurements show that defects with energy higher than 0.45 eV are not significantly participating in TSC emissions, dark current, and PC signals. This can be considered an interesting result: the prevailing of shallower defects on deep ones has been found to be beneficial on open circuit voltage and efficiency of other perovskite solar cells [12]. In the range of interest for solar cell operation, our study shows that both dark current and photocurrent are mainly dominated by energy levels in the range 0.40–0.45 eV, close to those assigned by literature to lead-related defects [10]. Comparing our results with those presented in the recent literature [20] on CsPbBr_3_ crystals (TSC analyzed with SIMPA) we observe that, in the literature, much higher capture cross-sections are assumed, in the range 10^−18^–10^−21^ m^2^. Hence, for the same energy level, TSC emission occurs at a much lower peak temperature. Moreover, a higher capture cross-section gives rise to a narrower peak so, for a single TSC peak that in principle could be discussed in terms of a single emission, in [20], it is necessary to consider the involvement of several emissions from very different energy levels. In our work, using the Chen method, we could first evaluate the order of magnitude of the energy level and then estimate a cross-section in the order of 10^−26^ m^2^, much lower than what is assumed in [20]. Then, β-variation and delayed-heating methods allowed us to better distinguish between single and multiple emissions, finally demonstrating the existence of two quasi-continuous energy level distributions in the ranges 0.11–0.27 eV and 0.42–0.45 eV.

## 6. Conclusions

Thermally stimulated current (TSC) tests have been carried out in the temperature range 100–400 K with microcrystalline CsPbBr_3_ films deposited, by drop-casting, on alumina printed circuit boards. Cross-correlating results obtained using different methods (Chen, heating-rate variation, and delayed-heating methods) we have been able to estimate parameters such as energy level, capture cross-section, and concentration of the involved defects. Very broad TSC emissions are interpreted as due to very low capture cross-sections, about 10^−26^ m^2^. Trap densities are in the range 10^13^–10^16^ cm^−3^. A set of energy levels in the range 0.11–0.27 eV, active below room temperature, suggests a quasi-continuum distribution of states with almost constant density. Above room temperature, up to 400 K, TSC is dominated by broad emission involving defects with energy levels in the range 0.42–0.45 eV. Dark current appears to be thermally activated in the whole investigated temperature range by an energy around 0.40 eV. Photocurrent transients at room temperature are well explained in terms of capture/release processes involving an energy level at 0.45 eV, in good agreement with TSC results.
